# Clusters of Sudden Unexplained Death Associated with the Mushroom, *Trogia venenata,* in Rural Yunnan Province, China

**DOI:** 10.1371/journal.pone.0035894

**Published:** 2012-05-17

**Authors:** Guo-Qing Shi, Wen-Li Huang, Jian Zhang, Hong Zhao, Tao Shen, Robert E. Fontaine, Lin Yang, Su Zhao, Bu-Lai Lu, Yue-Bing Wang, Lin Ma, Zhao-Xiang Li, Yang Gao, Zhu-Liang Yang, Guang Zeng

**Affiliations:** 1 Chinese Field Epidemiology Training Program, Chinese Center for Disease Control and Prevention, Beijing, China; 2 Yunnan Institute of Endemic Diseases Control and Prevention, Dali, Yunnan Province, China; 3 Cardiovascular Institute and Fuwai Hospital, Chinese Academy of Medical Sciences and Peking Union Medical College, Beijng, China; 4 Centers for Disease Control and Prevention, Atlanta, Georgia, United States of America; 5 Kunming Institute of Botany, Chinese Academy of Sciences, Kunming, Yunnan Province, China; Jr., The University of Texas at San Antonio, United States of America

## Abstract

**Introduction:**

Since the late 1970's, time-space clusters of sudden unexplained death (SUD) in northwest Yunnan, China have alarmed the public and health authorities. From 2006–2009, we initiated enhanced surveillance for SUD to identify a cause, and we warned villagers to avoid eating unfamiliar mushrooms.

**Methods:**

We established surveillance for SUD, defined as follows: sudden onset of serious, unexplained physical impairment followed by death in <24 hours. A mild case was onset of any illness in a member of the family or close socially related group of a SUD victim within 1 week of a SUD. We interviewed witnesses of SUD and mild case-persons to identify exposures to potentially toxic substances. We tested blood from mild cases, villagers, and for standard biochemical, enzyme, and electrolyte markers of disease.

**Results:**

We identified 33 SUD, a 73% decline from 2002–2005, distributed among 21 villages of 11 counties. We found a previously undescribed mushroom, *Trogia venenata*, was eaten by 5 of 7 families with SUD clusters compared to 0 of 31 other control-families from the same villages. In *T. venenata*–exposed persons SUD was characterized by sudden loss of consciousness during normal activities. This mushroom grew nearby 75% of 61 villages that had time-space SUD clusters from 1975 to 2009 compared to 17% of 18 villages with only single SUD (p<0.001, Fisher's exact test).

**Discussion:**

Epidemiologic data has implicated *T. venenata* as a probable cause of clusters of SUD in northwestern Yunnan Province. Warnings to villagers about eating this mushroom should continue.

## Introduction

Since the late 1970's, clusters of sudden unexplained death (SUD) scattered throughout a 60,000 km^2^ area of northwest Yunnan, China [1], [2], alarmed the public, health authorities, and local governments. These SUD clusters occurred from late June through early September in remote hamlets most with over 2000 m altitude [1], [2] and the overall pattern showed strong time-space clustering [1]–[4]. Deaths in specific family or village groups occurred over a week, the highest risk was among young and middle aged adults and women, and all ethnic groups can be affected [1], [3]. Initial syncope and coma occurred primarily during diurnal activities and rarely while sleeping, fever and other signs of infection were rare [3]. Within family units the risk did not differ between genetically related and unrelated persons [3]. Postmortem examination of 29 cases did not reveal a common lesion; but mild, focal lymphocytic myocarditis, insufficient to explain sudden death, was seen in 38%, arrythymogenic right ventricular cardiomyopathy (ARVC), a chronic inherited condition, was seen in 14% [5].

This pattern was distinct from sudden unexplained nocturnal death (SUND) in nearby Southeast Asian countries and the Philippines. SUND primarily affects young adult males [6]–[9]. Deaths are usually nocturnal, during sleep, and diurnal deaths occur during rest. Time space clustering was not evident. Family clustering was uncommon and when it did occur it showed an inherited pattern with siblings dying in different places in different years [10], [11]. SUND has been shown to occur in persons with electrocardiograms that have a pattern typical of the Brugada syndrome [11]–[14]. A polymorphic genetic mutation is responsible for this conduction defect in Southeast Asians [15]–[17]. Several other inherited conduction and structural heart defects also cause sporadic sudden cardiac death including the long QT syndrome, ARVC, and catecholaminergic polymorphic ventricular tachycardia (CPVT) [18], [19]. Unlike SUNDS and these inherited defects, the pattern of Yunnan SUD pointed to brief exposure to an environmental agent.

In 2005, we launched a special surveillance system throughout previously affected counties. Field investigations done in 2005 indicated that the deaths were likely cardiac in origin and implicated picking or eating wild mushrooms as a probable common exposure. However, no specific mushroom species was linked to these sudden deaths.

In general, deaths from mushroom intoxication is preceded and accompanied by characteristic disease syndromes [20]. Amatoxins, cyclic peptides from several species of the genera *Amanita*, *Lepiota*, and *Galerina*, produce gastroenteritis followed from 2–6 days after ingestion by liver necrosis [21]–[23]. The low LD50 (<1 mg/kg) from the most toxic amatoxin reflect a fatal dose from eating as little as one mushroom, a case fatality rate of 10–15%, and a high incidence of hepatic failure among exposed persons [21], [22]. Orellinine from *Cortinarius* species causes acute renal failure that can lead to chronic renal failure [21], [22]. A case series in 1957 reported an 11% case fatality rate [21]. However, in more modern accounts, an 8% incidence of end-stage renal failure requiring hemodialysis or kidney transplantation has apparently replaced mortality [21]. Severe poisoning from *Gyromitra* mushrooms can result in hepatic failure, delirium, seizures, and coma [24]. Death occurs in 2% to 10% of *Gyromitra* poisonings. In 2004 in Japan, an epidemic of encephalopathy with 19 deaths occurred among patients with pre-existing chronic renal failure, many of whom had eaten *Pleurocybella porrigens* a few days to weeks before onset [25]. Further attribution of causation of the epidemic to this mushroom remains speculative. Fatalities from rhabdomyolysis following ingestion of *Tricoloma equestre* and *Rusella subnigrcans* have occurred, and three toxic cycloprop-2-ene carboxylic acids have been identified from *R. subnigricans* [25]–[28]. A few other mushroom toxins rarely cause death if eaten in large amounts particularly by young children [21], [24]. In contrast to these known causes of fatal mushroom poisoning, the sudden deaths in Yunnan did not display the characteristic premortem syndromes of these other mushrooms.

Accordingly, in 2006 we initiated intensified surveillance and follow-up of new SUDs to identify a specific toxic mushroom or a toxic substance (e.g. heavy metals) concentrated in several mushroom species. We report herein the epidemiology of SUD from 2006–2009 which focused subsequent control measures and toxicological studies on heretofore undescribed mushroom now known as *Trogia venenata* [29].

## Methods

### Surveillance

We established a surveillance system for timely detection of SUD throughout 22 previous affected counties. We defined SUD as any death after exclusion of known causes, in a person who had sudden loss of ability to carry out normal activity due to serious physical impairment with death following in <24 h. We defined a SUD cluster as ≥2 SUD occurring in a village within 30 days. We defined a mild case as any illness in the same household or nearby related households during one week before or after a SUD.

We trained staff of 22 county CDCs to apply the case definition, take required samples and data and report. Each county CDC in turn trained staff of township hospitals. Township hospital staff then trained village doctors. We required village and township doctors to report SUD consistent with our definition within 2 hours. Yunnan Bureau of Health also issued a document requiring all CDC in Yunnan at or above the county level to report SUD.

### Field investigation

For each detected SUD or SUD cluster we interviewed witnesses of the SUD, mild case-persons, well family members of SUD victims, and local doctors to collect the information about daily activities, exposure to mushrooms and other toxic or potentially toxic substances and symptoms and signs of illness. We also selected other asymptomatic villagers within 10 years of age of the SUD victim as control villagers. We asked them the same questions about exposures. We developed local mushroom photo album that included edible and toxic mushrooms and mushrooms of unknown toxicity. We used it to aid in determining the type of mushroom exposure during 2 weeks prior to the SUD of deceased persons, their family members, and control-villagers.

### Mushroom identification and reconnaissance

In 2006, we found that SUD victims at 2 widely separated villages had eaten the same mushroom before death. A mushroom morphological expert from Kunming Institute of Botany, Chinese Academy of Science (co-author YZL) identified the genus of this mushroom as *Trogia* and found that it did not belong to any previously described species of this genus. This mushroom is now established as a new species, *Trogia venenata* (Mycobank registration number MB 561711) [29]. At all subsequent village investigations mushrooms of this type were searched for and similarly identified. We initiated *T. venenata* reconnaissance in 61 villages affected with SUD clusters from 1975 to 2009, 18 villages with single SUD cases reported from 1975 to 2009, and 15 villages that did not report SUD from 1975 to 2009 but were from the same townships as villages with SUD clusters. Before June 2006, we visited all villages with previous SUD clusters and warned all villages not to eat unfamiliar mushrooms. In May, 2008, we repeated this warning using local names and descriptions of *T. venenata*.

### Biological specimens

We collected blood serum of suspect mild cases, family member (lived together 2 weeks prior to the SUD or in socially related villagers with recent activities in common) and villager-controls. Suspecting cardiac toxicity, we determined creatine kinase (CK), creatine kinase MB isoenzyme (CKMB), a-hydroxybutyrate dehydrogenase ( HBDH), cardiac troponin I (TNI), myoglobin (Mb/MYO), and gamma-glutamyltransferase (GGT). Since the cause of death was otherwise unknown, we also ran a panel of standard biochemical markers that would be relevant in the characterization of any illness of unknown cause. These markers included alanine aminotransferase (ALT), aspartate aminotransferase (AST), alkaline phosphatase (ALP), lactate dehydrogenase (LDH), total bilirubin (TBIL), direct bilirubin (DBIL), indirect bilirubin (IBIL), glucose (GLU), glycosylated serum protein (GSP/FMN), total protein (TP), albumin (ALB), globulin (GLB), albumin to globulin ratio (A/G); urea nitrogen (BUN), creatinine (CREA), uric acid (UA); total cholesterol (CHOL), high density lipoprotein cholesterol (HDLC), low density lipoprotein cholesterol (LDLC), triglycerides (TG); potassium (K), sodium (Na), chlorine (Cl), calcium (Ca) and phosphorus (P). All determinations were made using fully automatic biochemical analysis using HITACHI 7600-110/7170A biochemical analyzers in city or county hospitals.

### Pathology review

Licensed physicians performed autopsies of SUD cases. All pathology specimens were reviewed by pathologists from Cardiovascular Institute and Fuwai Hospital, Peking Union Medical College and Chinese Academy of Medical Sciences. Pathologists made gross observation of the organ surface and the cut plane. For the heart they also measured the left and right ventricular free wall thickness using a ruler (mm). For adults they also determined whether the lumen of the coronary arteries lumen was narrowed. The organs were then fixed with 10% formalin for at least 3 days. 12 heart tissue blocks (1.8 cm*3.0 cm) were taken from anterior, lateral and posterior walls of left ventricle and anterior and posterior walls of right ventricle. One tissue block was taken from each of the other organs. However, additional tissue blocks were taken from organs with changes on gross pathology. All tissue blocks were embedded in paraffin and tissue sections were cut at 5 um in thickness. 30 (mean) sections from each tissue block were mounted on slides and stained with haematoxylin and eosin according to standard methods [30]. For each slide we examined by light microscopy the entire area at 5 x and progressively increased magnification to 100x for selected areas including all that appeared abnormal at 5 x and some normal areas. Overall more than 80 fields at 100x fields were examined per section.

### Informed consent

Because villagers were illiterate, we obtained informed consent orally for blood collection from individuals and for autopsies from family members. The local village doctor explained the procedure and risks in the local language and the consenting person acknowledged with a fingerprint or for the few with minimal literacy, a written mark or character. Approval for oral consent and other aspects of this investigation was granted by the Ethics Committee of Chinese Center for Disease Control and Prevention (ID 200503). This investigation was also part of an ongoing response to a critical public health event.

### Data analysis

The X^2^ or Fisher's exact test was used to assess the significance of differences between subgroups by using Epi Info 3.5.1. In a retrospective cohort approach rates of SUD and symptomatic family members were compared between persons who ate and did not eat the suspect mushrooms. Mantel-Haenszel risk ratio and 95% confidence interval was calculated to adjust for family units. All tests of significance were 2-sided and P<0.05 was considered statistically significant.

## Results

### Surveillance and intervention

**Figure 1:**
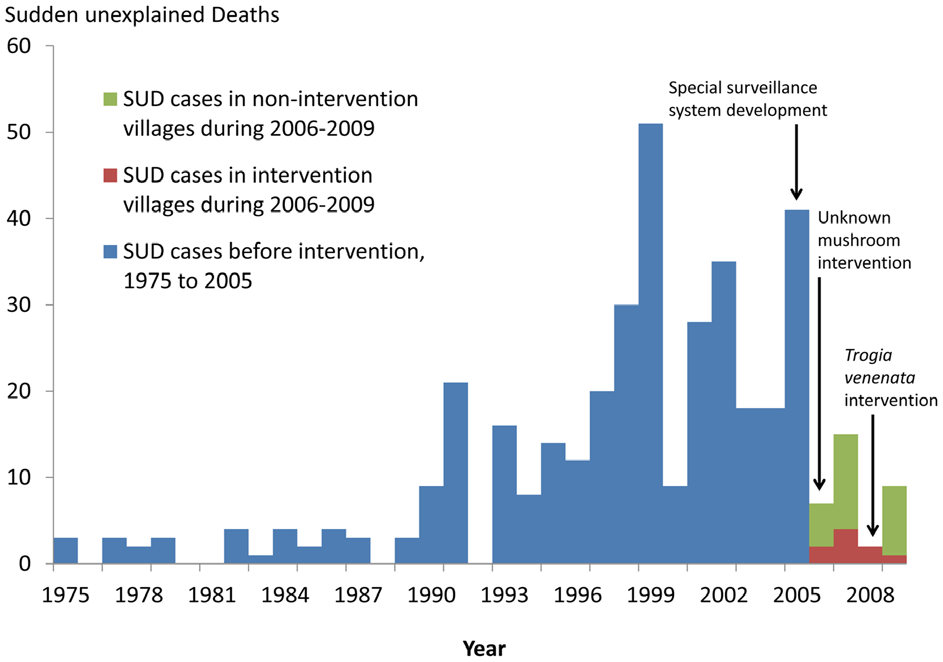
Distribution of 395 sudden unexplained deaths (SUD) in intervention area, 1975–2009, Yunnan Province, China. Deaths increase gradually by year to reach an average of 21 per year from 1990 to 2005. Following warnings in 2006 to villagers about eating unfamiliar mushrooms deaths decrease abruptly. A specific warning about *Trogia venenata* in 2008 is followed by a sustained decrease to near zero.

During June to September 2006–2009, the special surveillance system identified 33 SUDs and 17 SUD-associated, mild cases in 21villages of 11counties. This represented a 73% decline from the 121 SUD detected in the same surveillance area from 2002–2005 (Figure 1). Of 33 SUDs, 73% (24/33) of SUD were from villages that did not have SUD reported before 2006 and thus had not received warnings about avoiding unfamiliar mushrooms. Of the 33 SUDs, 18 plus 13 mild cases occurred in 7 time-space clusters in 8 villages (one cluster involved 2 villages) of 6 counties. The remaining 15 single SUDs plus 4 mild cases were from 14 villages in 9 counties (1 village had single SUDs in two different years). (Table 1).

**Table 1:** Clustered and single sudden unexplained deaths (SUDs) by *Trogia venenata* exposure from SUD surveillance in 22 counties, 2006–2009, Yunnan Province, China.

		*Trogia venenata* *		
	No.	Ate	Present	Total
Cluster	Events	5	6	7
	Involved villages†	6	7	8
	SUDs	9	16	18
	suspected mild cases	10	13	13
Single	Events	0	5§	15
	Involved villages	0	5	14‡
	SUDs	0	5	15
	suspected mild cases	0	4	4

* Trogia *venenata* ate means that at least one SUD victim in event ate *Trogia venenata*. *Trogia venenata* present means that this mushroom grew around the village.

† one cluster involved 2 villages.

‡ 1 village had single SUDs in 2007 and 2008

§ 4 villages also had SUD clusters before 2006.

In the 7 clusters, There was a mean of 2.6 SUDs (range: 2–4) and 1.9 suspected mild cases (range: 1–4) per cluster. All clusters were in small hamlets that were detached from main village. The 7 clusters involved 14 households with 72 persons and the attack rate for all illness (SUD and mild cases) was 43% (31/72), for SUD 25% (18/72) and for case fatality 58% (18/31). The median age of all illness (SUDs and mild cases) was 29 years (Range = 8–56), for SUD 39 years (Range = 8–56). Among all members of affected families the attack rate (AR) of all illness in females was 54% (21/39) compared to 30% (10/33) among males. (*P*<0.05).

### Exposures

**Figure 2:**
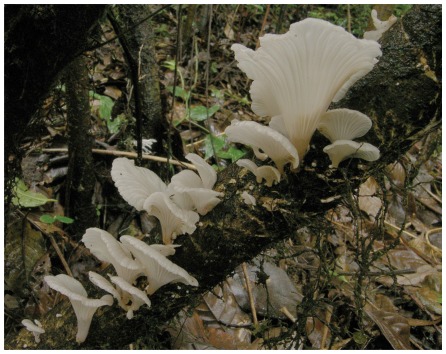
*Trogia venenata* from Tengchong County, Yunnan Province, China. Growing in characteristic natural environment at 2120 m, 102 42 E longitude; 25 04.007 N latitude. Scale of measurement in centimeters (Height = 5cm–10cm, width = 3cm–7cm).

**Figure 3:**
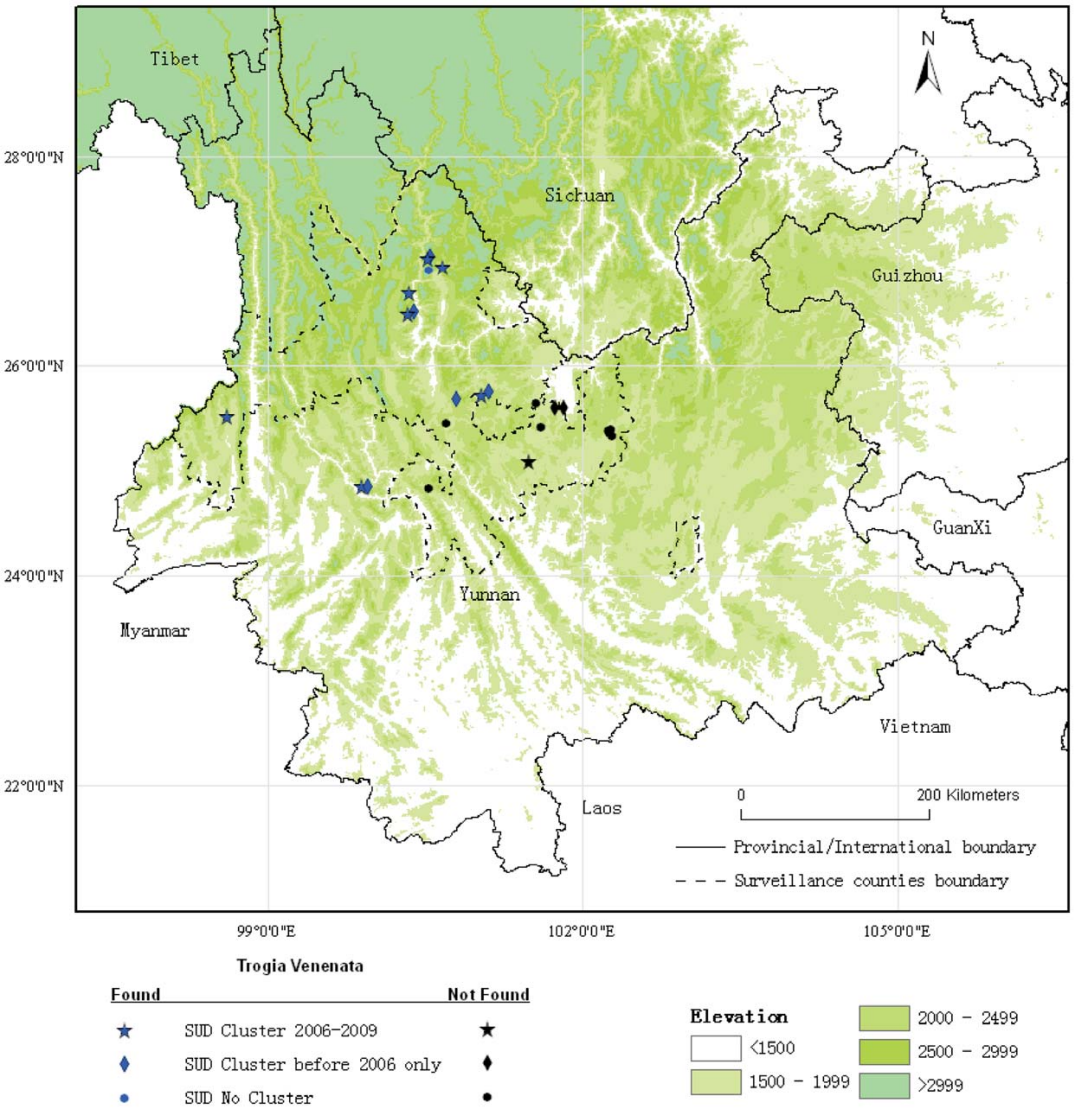
Sudden unexplained deaths (SUDs) by village and presence of *Trogia venenata*, 2006–2009, Yunnan Province, China. SUD clusters with the presence of *T. venenata* occur throughout the intervention area with the exception of the far eastern section. All affected villages are in mountainous terrain from 1800 to 2800 meters. One village outside the south central boundary of the area we also found *T. venenata* and clusters and single SUDs in different years.

During the field investigation of the first 2 SUD clusters (July 2006), we found that the victims had eaten a heretofore unspeciated mushroom, now named *Trogia venenata* (Figure 2) (Mycobank registration number MB 561711) [29]. This mushroom was delicate and flowerlike. It measured from 5–10 cm wide and 3–7 cm long. Its color ranged from pinkish, to dirty white to light brown. We found this mushroom in the north-central and northwest of the surveillance area at altitudes between 1800 and 2800 m (Figure 3). Finding it required 3–4 hours of searching, but, where found, it grew profusely on dead trees. Villagers in different counties used different common names for this mushroom in different local dialects. Some villagers would pick this mushroom if they ran across it while picking known, edible mushrooms for sale to commercial buyers. However, *T. venenata* had no commercial value, and the villagers could only eat it. Some reported that it caused diarrhea which they could avoid if they ate small amounts at a time or dried it first.

We found *T. venenata* growing nearby 7 of 8 villages that had SUD clusters during 2006–2009. (Table 1). We found this mushroom at 75% (46) of 61 villages that had SUD clusters reported from 1975 to 2009. This compares to 17% (3) of 18 villages that had only single cases during the same time period (p<0.0001, Fisher's exact test) and 33% (5) of 15 villages with no reports of SUD (p<0.0001, Fisher's exact test). All villages with SUD clusters before 2006 had been warned not to eat unfamiliar mushrooms in May 2006 and not to eat *T. venenata* in May 2008. Only 1 of the 7 villages with a SUD cluster from 2006–2009 had a previous SUD cluster and had been thus warned.

At the 7 current clusters in surveillance area, victims had exposure to *T. venenata* at 6. (Table 1). In 5 clusters witnesses (including family members or mild suspect case-persons) reported that the victims had eaten the mushroom. We obtained a history of eating this mushroom in 5 clusters. In the sixth cluster we found a substantial amount of *T. venenata* in the kitchen, however, the only survivors were two young children who could not tell us if they or their deceased family members had eaten the mushrooms. At the seventh remaining cluster, at a village where we did not find *T. venenata*, we found ARVC on the autopsy of both victims, a married couple.

The 6 SUD clusters with exposure to *T. venenata* occurred in 7 hamlets with 232 households and 1076 population. 16 SUDs and 13 suspected mild cases occurred in 13 household with 69 family members. 92% (12) household had exposure to *T. venenata* except one household with single mild cases. This compares to no exposure to *T. venenata* in the 2 weeks prior to the SUD event in 31 control-households (p<0.001, Fisher's exact test). We also did not identify any other potential toxins such as pesticides, rat poisons, chemicals, drugs, and other wild plants, fermented corn flour, moldy or spoiled grain, and traditional medicine more frequently in case-households than control households.

Of the 5 clusters with a definite history that *T. venenata* was eaten, there were 59 family members. From our interviews we found that 30 persons ate *T. venenata* and 29 did not eat this mushroom. Of 30 exposed, 63% (19) developed illness including 30% (9) SUD. Among the 29 family members without histories of eating *T. venenata*, 17% (5) developed illness including 10% (3) with SUD. After adjusting (Mantel Haentzel test) by household, the relative risk for disease was 3.9 (95% CI = 1.5–10). Excluding persons with mild illness the relative risk for SUD was 2.3 (95% CI = 0.67-8.1). Estimates of the amount of *T. venenata* eaten by 3 SUD victims were 100g (once) in a woman with ARVC and 400 g and 1500 g total eaten several times over 1 week. Among all SUD cases the median time from last eating *T. venenata* to death was 4 days (range 16 hr to 15 days).

### Characteristics of illness and death associated with *Trogia venenata*

Among the 13 SUD cases with exposure to *T. venenata*, 12 of the deceased were previously in good health while 1 had mild chronic complaints (dizziness and fatigue). One person died while sleeping. The other 12 suddenly lost consciousness during daily activities and either died within 10 minutes (7) or became comatose and died within a day (5). While unconscious 2 had convulsions, 2 vomited, and 1 had urinary incontinence. Just preceding the acute, fatal episode, 7 had one or more episodes of syncope and dizziness. For 3–5 days before this acute, fatal episode, 5 had a variety of transient, recurrent symptoms that did not interfere with their daily activities (Table 2). In 4 of these SUD clusters 10 extended or immediate family members of the deceased also had mild transient symptoms. Compared to SUD cases, they had palpitations and chest distress more frequently.

**Table 2:** Symptoms among 13 SUD and 10 mild cases associated* with *Trogia venenata* during 2006–2009, Yunnan, China.

	Number		Percent		
Symptoms	Mild	SUD	Mild	SUD	p-value†
Palpitation	8	2	73	15	**0.01**
Dizziness	6	3	55	23	NS‡
Chest distress	6	0	55	0	**0.003**
Shortness of breath	4	2	36	15	NS
Abdominal pain	5	6	45	46	NS
Syncope or temporary loss of consciousness	4	8	36	62	NS
Headache	5	3	45	23	NS
Cough	4	0	36	0	**0.03**
Vomiting	4	4	36	31	NS
Sore throat	3	3	27	23	NS
Convulsion	1	3	9	23	NS
Chest pain	2	3	18	23	NS
Fatigue	1	3	9	23	NS
Hand and foot numbness	1	1	9	8	NS
Shoulder or neck pain	1	3	9	23	NS
Coma	0	6	0	46	**0.02**
Difficulty breathing	0	1	0	8	NS
Nausea	0	1	0	8	NS
Diarrhea	0	1	0	8	NS

* Member of a group that ate T. venanata.

† p-value for comparison of frequency of symptom in SUD and mild cases by Fisher's exact test.

‡ not statistically significant, p>0.05 Fisher's exact test.

**Figure 4:**
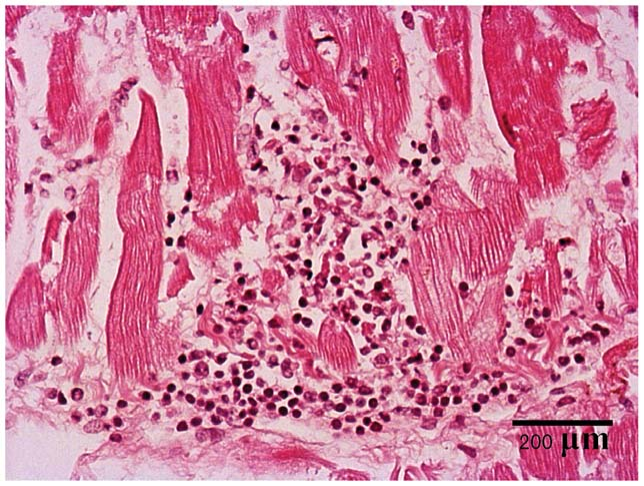
Heart muscle section from the right ventricle from a sudden unexplained death case. Heart muscle section from the right ventricle from a SUD case, 100x, hematoxylin-eosin, showing focal lymphocytic infiltrates and dissolution and breaking of muscle fibers.

We did postmortems on 4 SUD cases with exposure to *T. venenata*. All had microscopic, localized dissolution, disruption, or necrosis of heart muscle fibers with focal lymphocytic infiltration (Figure 4). We noted the additional acute lesions: localized lymphocytic infiltration of the liver in 3, pulmonary alveolar edema in 2, acute kidney tubular necrosis, hepatocyte necrosis, and congestion of the liver, of the lung, and of the spleen in one each. We found the following underlying conditions in one patient each: ARVC, a patent foramen ovale, and 75% stenosis of the artery of the atrioventricular node. Brain and skeletal muscle were not examined.

The comparison serum biochemistries, enzymes, and electrolytes between of suspected mild case who shared *T. venenata* meals with SUD victims to village control persons revealed a few notable differences (Table 3). Creatine kinase and the MB isoenzyme, myoglobin, and AST ranged from 1.6 to 3.8 times higher than village controls. These differences were statistically significant only for AST. Serum protein was decreased relative to control-villagers. ALT and other indicators of liver function did not differ. Two suspected mild cases were hypoglycemic (3.31 and 3.65 mmol/L). Serum sodium was higher (mean = 143.3 mmol/L) than in control-villagers (mean = 140.2 mmol/L) but none were hypernatrimic. There were no differences or abnormalities of serum potassium.

**Table 3:** Selected clinical chemistry determinations from serum of persons with mild cases and other villagers (controls) 6 villages with clusters of sudden unexplained death, 2006–2009, Yunnan Province, China.

	Number		Mean value		RD †	Percent elevated		Percent decreased	
Determinations	Mild*	Villager	Mild	Villager		Mild	Villager	Mild	Villager
Creatine kinase (U/L), CK	9	80	575	188	3.0↑	44	29	-	-
Creatine kinase MB isoenzyme (U/L), CKMB	6	63	60	29	2.1↑	67	41	-	-
Myoglobin (µg/L), Mb/MYO	3	27	80	21	3.8↑	33	0	-	-
Cardiac troponin I(ng/ml), TNI	1	24	0.01	0.01	1.0	0	0	0	0
Alanine aminotransferase (U/L), ALT	9	73	43	27	1.6↑	22	4.1	-	-
Aspartate aminotransferase (U/L), AST	9	80	53	26	2.0↑‡	44 §	3.8		-
Lactate dehydrogenase(U/L), LDH	8	80	287	228	1.3↑	75	44	-	-
Glucose (mmol/L), GLU	10	51	5.2	4.8	1.1↑	10	3.9	20§	0
Glycosylated serum protein, GSP/FMN	3	24	1.3	1.8	1.4↓‡	0	0	33	0
Total protein (g/L), TP	9	56	66	78	1.2↓‡	0	27	22§	0
Albumin (g/L), ALB	9	56	43	47	1.1↓	0	0	11	0
Globulin (g/L), GLB	9	34	23	30	1.3↓‡	11	8.8	22	12
Sodium, meq/L	12	80	143	140	1.0↑	25§	0	8.3	1.3
Potassium, meq/L	12	80	4.1	4.4	0.9↓	0.0	3.8	0.0	2.5
Calcium, meq/L	12	72	2.5	2.5	1.0	17§	0.0	0.0	1.4

* 2 unknown and 1 not admitted exposure to *T. venenata* for creatine kinase, alanine aminotransferase, aspartine aminotransferase, glucose, total protein, albumin, globulin, and Na^+^. 2 unknown for creatine kinase MB isoenzyme, 1 unknown for myoglobulin, 1 unknown and 1 not admitted for glycosylated serum protein.

† RD (Relative difference =  (mean value for mild cases)/(mean value for control villagers). Relative increase↑, relative decrease↓ between mild cases and control-villagers.

‡ p<0.05, Kruskal Wallis test between mild cases and control-villagers.

§ p<0.05, Fisher's exact test between mild cases and control-villagers.

## Discussion

The data from intensive surveillance and intervention for SUD in northwestern Yunnan Province, China, has revealed an association of clusters of SUD with a newly described mushroom species, *T. venenata*. This association was supported at the village level with a high frequency of this mushroom in villages with SUD clusters. Support also came from the household level revealing higher frequency of *T. venenata* in households with SUD clusters compared to other houses in the same villages. Finally within exposed families we found the same association comparing persons who ate and did not eat the mushroom. Also supporting our observation is the major, extended decrease in SUD that followed public warnings about eating unfamiliar mushrooms (2006–2007) and *T. venenata* (2008–2009). In any investigation of SUD, background cases from a variety of causes will be present. Accordingly, some SUD and particularly many of the single cases could have been from background cardiac causes such the ARVC, long QT, and Brugada syndrome [18], [19]. Also these underlying defects could also have increased the susceptibility of villagers to *T. venenata* poisoning.

The characteristics of the fatal *T. venenata* exposures reinforce our earlier impression of the distinctiveness of this syndrome from those seen with other mushrooms. Specifically, persons exposed to *T. venenata* were either asymptomatic or had mild transient symptoms before losing consciousness and dying. Other fatal mushroom poisonings have characteristic syndromes of progressing severity and often incapacitation over one or more days before death [18]. Amatoxins result in a delayed and progressive onset of hepatic failure sometimes accompanied by renal failure [21]–[23]. Orellanine leads to renal failure which often becomes chronic [21], [22], [24]. Fatal gyromitrin poisoning is preceded by hepatic failure, delirium, seizures, and coma [24]. Two species of mushrooms, *Russula subnigricans* and *Tricholoma equestre* can produce rhabdomyolysis characterized by muscle stiffness, pain, and tenderness; and massive elevations in CK [25]–[28]. Deaths may ensue in severe cases from damage to the heart. Although we considered heart damage from rhabdomyolysis as a possible cause of sudden death, we did not find typical symptoms of rhabdomyolysis and elevations in CK were clinically and statistically insignificant.

Our epidemiologic observations have led to toxicological research which has identified from *T. venenata*, 2 amino acids (2*R*-amino-4*S*-hydroxy-5-hexynoic acid and 2*R*-amino-5-hexynoic acid) that differ by one hydroxyl group [31]. Both are fatally toxic to mice. One of these amino acids was identified in the postmortem heart blood from a single, isolated, SUD from a village where *T. venenata* grew and where SUD clusters occurred before 2006. The experimental animal studies have one key similarity to the human cases. In mice a 1.6 fold increase in CK was seen after 3 daily sub-lethal doses while CK and other muscle-related enzymes (myoglobin, and AST) were elevated in family contacts of SUD cases that ate *T. venenata* [31]. However, the experimental study examined only CK.

Normally, indigenous mushroom gatherers are aware of and avoid local toxic species of mushrooms. Several factors may have contributed to the ignorance of the danger from *T. venenata*. This type of mushroom poisoning was previously unknown. The symptoms between eating the mushroom and sudden death reported from only 50% of fatalities, were very mild, and showed no tendency to worsen or progress prior to the sudden death. Individual villagers had past experience of eating this mushroom with only minimal and tolerable ill effects. This mushroom was hard to find and had no commercial value thus lowering the chance that it would come to the attention of outsiders. Since each village named the mushroom differently, a village that did recognize the danger would not communicate this fact clearly to other villages. We also could surmise that among the villages in NW Yunnan where *T. venenata* grew, some did recognize *T. venenata* as a dangerous mushroom and did stop eating it.

Despite our best attempts to reach the sites promptly, clinical data was very incomplete. We were invariably too late to obtain key clinical tests and specimens from SUD victims before death. Postmortem specimens were obtained on a few cases and because of cultural taboos did not include brain. Differences in symptomatology between mild and SUD cases could have resulted simply because the deceased did not complain to family or friends of all symptoms before losing consciousness. In comparison complete symptom histories could be obtained from living persons who had mild cases through face-to-face interviews. The history of eating this mushroom may have sometimes not been disclosed. In particular, in villages with prior SUD clusters eating the mushroom was tantamount to admitting non-compliance with official warnings. Indeed, a collaborator identified the toxic amino acid in the postmortem heart blood from a single SUD case for which family members denied that the deceased was exposed to *T. venenata* [31]. Our data on the quantity and timing of the exposure to *T. venenata* was very sparse and had excessive variability in amount and frequency of exposure. Animal experiments will need to model moderate dose exposures over several days with assessment of changes in key biochemical indicators such as glucose, lipids, creatine kinase, troponins, and myoglobin.

We conclude that *T. venenata* is a strong suspect for a cause of clusters of SUD in Yunnan Province. Our findings were sufficient to institute targeted warnings to villagers throughout affected area. Continuing intensive surveillance during the 2010 and 2011 SUD season (June-September) identified 3 SUDs without exposure to *T. venenata* and no clusters of SUD in this intervention area. This apparently successful public health intervention underscores our finding. However, additional insights into the mechanisms of toxicity of *T. venenata* will most likely come from animal experimentation using the two implicated amino acids.
